# A Particular Case of Myocardial Injury

**DOI:** 10.3390/diagnostics15243136

**Published:** 2025-12-09

**Authors:** Dario Catapano, Antonia Ascrizzi, Luigi Falco, Santo Dellegrottaglie, Alessandra Scatteia, Francesco Saverio Loffredo, Enrica Pezzullo, Rita Gravino, Paolo Golino, Emilio di Lorenzo, Daniele Masarone

**Affiliations:** 1 Cardiology Unit, Department of Translational Medical Sciences, University of Campania “Luigi Vanvitelli”, 80131 Naples, Italy; dariocat90@gmail.com (D.C.); paolo.golino@unicampania.it (P.G.); 2 Advanced Cardiovascular Imaging Unit, Clinica Villa dei Fiori, 80011 Acerra, Italya.scatteia@gmail.com (A.S.); 3 Department of Cardiology, AORN dei Colli Monaldi Hospital, 80131 Naples, Italy

**Keywords:** eosinophilic myocarditis, myocardial injury, multimodality imaging, endomyocardial biopsy

## Abstract

We report a case of a patient admitted to our coronary intensive care unit with chest pain and elevated cardiac troponin. Coronary angiography showed no obstructive coronary artery disease. The patient had a history of chronic rhinosinusitis and nasal polyps, treated with dupilumab. Laboratory tests revealed marked hypereosinophilia, prompting a cardiac MRI, which showed findings consistent with eosinophilic myocarditis. This diagnosis was later confirmed by endomyocardial biopsy. This case highlights the importance of differential diagnosis in cases of myocardial injury (characterized by increased cardiac troponin) and underscores the value of CMR as the most accurate non-invasive technique for detecting myocarditis.

**Figure 1:**
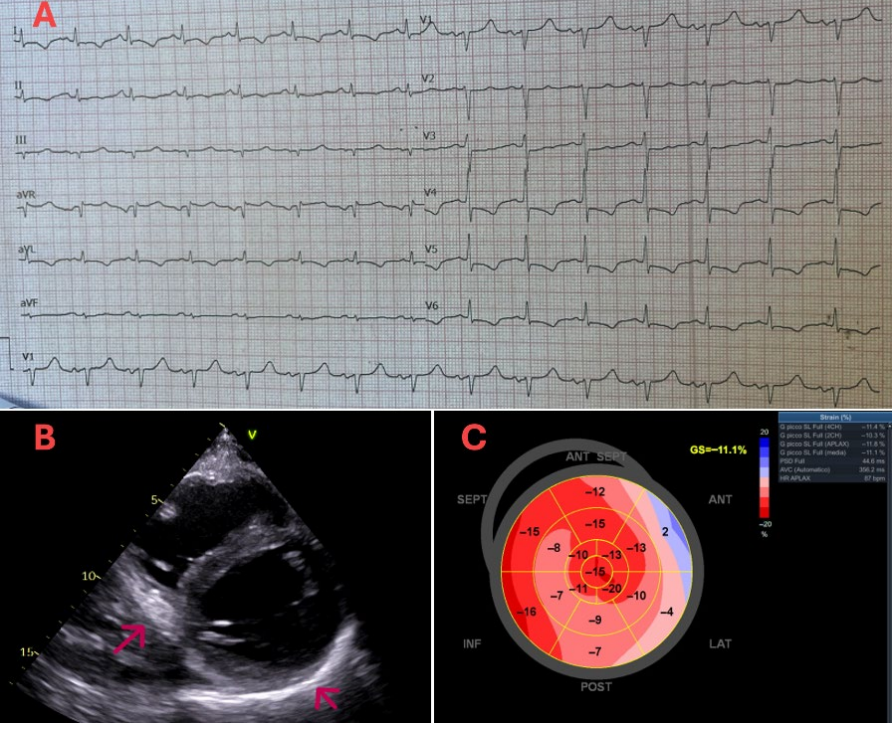
A 55-year-old man with a history of chronic rhinosinusitis with nasal polyps (CRSwNP), treated with dupilumab for the past 9 months, was admitted to our intensive care unit due to chest pain. Upon admission, his tests showed elevated high-sensitivity troponin I (5100 pg/mL), NT-proBNP (11,949 pg/mL), C-reactive protein (25 mg/L), and hypereosinophilia (2500/uL). Electrocardiogram revealed widespread ventricular repolarization abnormalities, including 1 mm ST-segment elevation in leads aVR and V1, along with diffuse ST-segment depression and T-wave inversion in other leads (Panel (**A**)). Transthoracic echocardiography showed a non-dilated left ventricle with diffuse hypokinesia, more pronounced along the inferior wall, and a left ventricular ejection fraction of 45% (Supplementary Video S1). Additionally, in the short-axis view at the mid-ventricular level, increased echogenicity and thickening of the parietal pericardium (red arrows), indicative of pericardial inflammation, were observed (Panel (**B**)). Finally, speckle tracking echocardiography (Panel (**C**)) demonstrated a moderate reduction in global longitudinal strain (−11.1%). Suspicion of acute coronary syndrome prompted coronary angiography, which revealed no obstructive coronary lesions, resulting in a diagnosis of myocardial injury.

**Figure 2:**
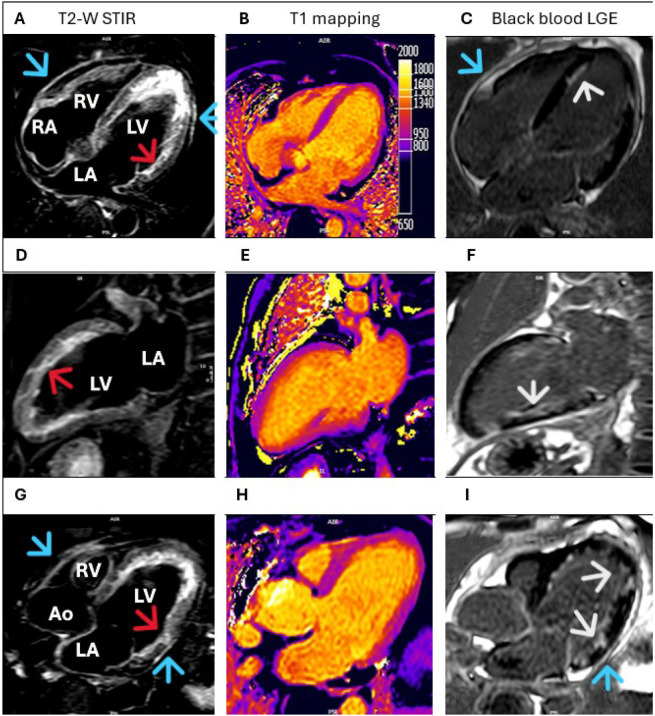
To examine the cause of myocardial injury, cardiac magnetic resonance imaging (CMR) was performed. STIR sequences (panels (**A**,**D**,**G**), red arrows) showed subendocardial and transmural myocardial hyperintensity, indicating edema. T1-mapping sequences (panels (**B**,**E**,**H**)) revealed increased native myocardial T1 values, represented on the color scale. Black blood late gadolinium enhancement (LGE) sequences (panels (**C**,**F**,**I**)), which improve contrast between the ventricular cavity and endocardium, demonstrated diffuse endocardial hyperintensity (white arrows), suggesting non-ischemic fibrosis. Additionally, pericardial hyperintensity was noted in the STIR (panels (**A**,**D**,**G**), blue arrows) and LGE (panels (**C**,**I**), blue arrows) sequences, indicating pericardial inflammation. These findings, support a diagnosis of eosinophilic myocarditis with acute pericarditis.

**Figure 3:**
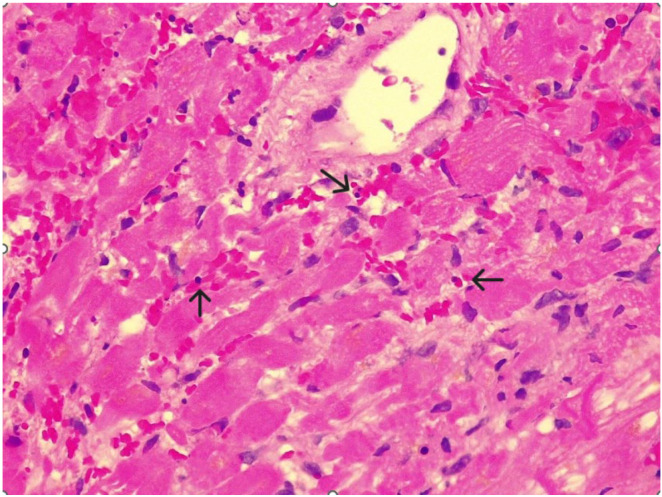
To confirm the suspected diagnosis of eosinophilic myocarditis, an endomyocardial biopsy (EMB) was performed. The results revealed myocardial fibers arranged irregularly (bright pink), with some showing signs of degeneration and disorganization. A dense interstitial inflammatory infiltrate composed of mononuclear cells and numerous eosinophilic granulocytes—easily identified by their bright pink cytoplasm (black arrows)—was present between the fibers. Certain areas displayed zones of myocyte necrosis, characterized by the loss of transverse striations and cytoplasmic disintegration. Additionally, a small intramyocardial vessel containing erythrocytes within its lumen was observed. The histopathological findings supported a diagnosis of severe; active necrotizing myocarditis. For the differential diagnosis of other hypereosinophilic syndromes, no involvement of other peripheral organs was observed beyond the cardiac manifestations, and parasitological and toxicological tests were unremarkable. Consequently, dupilumab was promptly discontinued. Treatment was initiated with high-dose corticosteroids (intravenous methylprednisolone at 1000 mg daily for three days, followed by a tapered course of oral prednisone) and mepolizumab (300 mg subcutaneously every four weeks). The patient responded rapidly at clinical, laboratory, and imaging levels, achieving long-term remission. In particular, at six months follow-up, the disease had stabilized, with no evidence of inflammation on CMR and normalization of the eosinophil count. Mild myocardial dysfunction persisted (LVEF 48%). Dupilumab is a monoclonal antibody targeting the alpha subunit of the interleukin-4 receptor and is approved for treating asthma, atopic dermatitis, and chronic rhinosinusitis with nasal polyps (CRSwNP). It may cause transient hypereosinophilia, and although organ involvement is uncommon, it requires careful investigation and monitoring because it can lead to serious or fatal complications [1]. Eosinophilic myocarditis (EM) is a rare form of myocarditis characterized by eosinophilic infiltration of the myocardium, often presenting in a fulminant course with rapid deterioration of left ventricular ejection fraction, and carries a high risk of malignant arrhythmias and thromboembolic events. EM diagnosis can be confirmed via EMB, which demonstrates eosinophilic infiltration, or through CMR showing characteristic findings along with peripheral eosinophilia. Treatment usually involves intravenous corticosteroids, sometimes combined with interleukin-5 inhibitors like mepolizumab [2]. This case emphasizes the importance of differential diagnosis in myocardial injury cases, where CMR plays a key role. In our case, CMR revealed signs indicative of eosinophilic myocarditis, prompting further investigation with EMB, which confirmed the diagnosis. A recent study on CMR in histologically confirmed eosinophilic myocarditis identified key features such as myocardial edema with apparent left ventricular hypertrophy, moderate to severe systolic dysfunction of an often-non-dilated left ventricle, multifocal and predominantly subendocardial myocardial LGE, and pericardial effusion [3]. Patients with conditions like chronic rhinosinusitis and nasal polyposis who are considered for dupilumab should undergo thorough screening for hypereosinophilic syndromes. During treatment, continuous clinical and laboratory monitoring is crucial. If suspicions arise, CMR is an essential non-invasive tool to screen for eosinophilic myocarditis, guiding subsequent biopsies that provide a definitive diagnosis. Early and accurate identification allows for prompt targeted immunosuppressive therapy, delivering significant short- and long-term benefits.

## Data Availability

The original contributions presented in this study are included in the article. Further inquiries can be directed to the corresponding author.

## Supplementary Materials

The following supporting information can be downloaded at: https://www.mdpi.com/article/10.3390/diagnostics15243136/s1, Video S1: Apical four-chamber, two-chamber and three-chamber along with a parasternal short-axis view at the mid-ventricular level cine acquisitions.
